# A transition to sustainable ocean governance

**DOI:** 10.1038/s41467-020-17410-2

**Published:** 2020-07-17

**Authors:** Tanya Brodie Rudolph, Mary Ruckelshaus, Mark Swilling, Edward H. Allison, Henrik Österblom, Stefan Gelcich, Philile Mbatha

**Affiliations:** 1 Centre for Complex Systems in Transition, University of Stellenbosch, Stellenbosch, South Africa; 2 The Natural Capital Project, Woods Institute for the Environment, Stanford University, Stanford, CA USA; 3 School of Public Leadership, University of Stellenbosch, Stellenbosch, South Africa; 4 Nippon Foundation Ocean Nexus Program, Earthlab, University of Washington, Seattle, WA USA; 5 WorldFish, Penang, Malaysia; 6 Stockholm Resilience Centre, Stockholm, Sweden; 7 Center of Applied Ecology and Sustainability, Department of Ecology, Pontificia Universidad Católica de Chile, Santiago, Chile; 8 Department of Environmental and Geographical Science, University of Cape Town, Cape Town, South Africa

**Keywords:** Ecology, Socioeconomic scenarios, Sustainability, Ocean sciences

## Abstract

Human wellbeing relies on the Biosphere, including natural resources provided by ocean ecosystems. As multiple demands and stressors threaten the ocean, transformative change in ocean governance is required to maintain the contributions of the ocean to people. Here we illustrate how transition theory can be applied to ocean governance. We demonstrate how current economic and social systems can adapt to existing pressures and shift towards ocean stewardship through incorporation of niche innovations within and across economic sectors and stakeholder communities. These novel approaches support an emergent but purposeful transition and suggest a clear path to a thriving and vibrant relationship between humans and the ocean. Oceans provide important natural resources, but the management and governance of the ocean is complex and the ecosystem is suffering as a result. The authors discuss current barriers to sustainable ocean governance and suggest pathways forward.

## Abstract (web summary)

Oceans provide important natural resources, but the management and governance of the ocean is complex and the ecosystem is suffering as a result. The authors discuss current barriers to sustainable ocean governance and suggest pathways forward.

## Introduction

A new relationship between humanity and the ocean is required to secure the continuity of the diverse life support roles provided by the sea^1,2^. Ocean governance faces the challenge of reflecting the multi-dimensional and interconnected role that the ocean plays in environmental health, economic prosperity and human well-being^1–4^, including justice and equity^1,5–7^.

The ocean, when considered as a whole, cannot be defined solely as either a state-governed public good, nor as a commodity or private good. The *World Commission on Environment and Development* states that “Oceans are marked by a fundamental unity from which there is no escape. Interconnected cycles of energy, climate, marine living resources, and human activities move through coastal waters, regional seas, and the closed oceans”^8^. The increasing prevalence and dominance of transnational corporations is also challenging the central role of governments in governance. Ocean governance thus needs a transformative shift from a state-centric approach to a global approach^9^ that takes into account the embeddedness of the ocean and associated actors in the wider planetary system^8,10^. It is therefore best seen as a commons (see Glossary in Table 1)—a non-state, non-private shared resource that can only be protected if stakeholders who depend on it take collective responsibility for preservation and restoration^11^ with self-devised protocols, values and norms^12^. An integrated approach will mean a transition to an adaptive, responsive global governance system (defined in Glossary in Table 1) for governing the ocean-as-commons; an approach which “does justice to humanity’s obligations to itself, and to the planet which is its’ home” (International Court of Justice Judge Weeramantry in Gabickovo-Nagymaros case 1997)^13^.

**Table 1:** Glossary of terminology.

Commons	A non-state, non-private shared resource, plus a defined community that devises protocols, norms and values to manage it (eg. Earth’s atmosphere)^33,42^
Environmental stewardship	Actions taken by individuals, groups, or networks of actors to protect, care for, or responsibly use the environment in pursuit of environmental and/or social outcomes in diverse social and ecological contexts^63^
Landscape pressures	Fundamental system conditions typically exhibiting gradual changes (e.g., demographics, resource depletion, climate change, technological innovation, urbanization, etc.) that synergistically and incrementally lead to shifts in the state of the environment and impacts on valued parts of ecosystems or on society
Generative ownership	Categories of private ownership which generate beneficial outcomes for common good^117^
Legal regime	A legal framework comprising principles and rules governing human activities or processes (eg. UNCLOS)
Meta-governance	Governance of governance among interacting groups^69^
Niche innovations	Novel approaches through which sectors or stakeholder communities interact with or produce goods from a social-ecological system in response to landscape pressures (eg. emergence of local renewable energy systems as an alternative to fossil fuels)
Regime (also socio-technical regime)	A tightly knit combination of regulations, key operators that produce products or services, consumers who depend on those products/services, the revenues that governments/agencies/regulators extract in the form of levies/taxes etc, the financial institutions who provide debt/equity, plus a substantial infrastructure operated by people who have been trained over decades to understand and operate the system in certain ways (eg. fossil fuel-based energy system)^118^
Reflexive governance	When the foundations of governance (the concepts, practices and institutions by which societal development is overseen) are questioned, and more relevant and effective alternatives are reinvented to reshape those foundations
Polycentric governance	A system of decision making in which multiple governing bodies interact to make and enforce rules within a specific policy arena or location
Volitional governance	Voluntary commitments aimed to deliver outcome-oriented activities^84^ eg. Nationally Determined Contributions under the Paris Agreement, the Voluntary National Review process set up under the United Nations SDG review mechanism^70^, and voluntary commitments under the Our Ocean Conference series (Registry of Voluntary Commitments UN Our Ocean Conference).

These global and intertwined dynamics^14^ are not fully reflected in the current legal definition in the United Nations Convention on the Law of the Sea (UNCLOS) and international customary law. The ocean comprises a global commons in Areas Beyond National Jurisdiction of any nation-state’s authority (the high seas and the seabed beyond continental shelves; Art 89 UNCLOS) as well as areas within nation-state sovereignty (maritime zones of coastal states including the Territorial Sea Art 2, 3, Exclusive Economic Zone Art 55, and Continental Shelf Art 76 (UNCLOS)). Governance of the Areas Beyond National Jurisdiction is generally weaker than within national jurisdictions^15^. A treaty for Biodiversity Beyond National Jurisdiction is being negotiated under the auspices of the United Nations^16^. Until it is finalised, more than 40% of the surface of the Earth has limited formal legal protection for its natural habitat and functional ecosystems (except through a patchwork of sectoral organisations)^6,17^.

The rationale for a sustainable ocean transition is becoming increasingly urgent^1,18^. If we do not act to change course, the ocean’s key biophysical functions could collapse^1^. Wider global and regional pressures (what we refer to as ‘landscape pressures’—see Glossary in Table 1) on the ocean include rising levels of greenhouse gas emissions^1,19^, changes in chemistry, which impact species and food webs throughout ocean ecosystems^2,8,20^, warming^21–23^, deoxygenation^24^, overfishing, and run-off of pollution from land and coastal sources^1,25^. The Earth system—and the ocean in particular—is at risk of “irreversible or unimaginable” change^26^.

In addition, the ocean is becoming a new economic frontier for production of energy, minerals and food^6^. Coastal zones are not only at the forefront of transition challenges, but they bear the brunt of climate change impacts^27^. Ocean sustainability transitions are therefore interdependent with those on land. The coastal zone serves as the interface between land-based society and expanded ocean economic activity. Coastal land use planning and integrated ocean management are therefore critical elements of a transition to a sustainable ocean economy^28^.

The ocean has been identified as one of six key coupled social–ecological systems that require transformative change to achieve the UN sustainable development goals^29^. Here we argue that a purposeful transition to a more sustainable ocean system requires a profound departure from business-as-usual to a global regulatory effort to pursue ocean sustainability. Transformation to a thriving ocean system requires changes in governance across sectors and scales, with effective and inclusive participation by multiple actors^10,30^. The end result would be a form of “polycentric governance” (see Glossary in Table 1) that can manage shared resources and ocean space^31^. Ocean polycentricism may require a rule-setting global institution (such as an Ocean Agency, Box 1), to support multiple governing bodies by establishing a shared vision, and creating principled guiding frameworks and processes to facilitate coherent systems-oriented regulation (^10^. In a complex world characterized by nationalist resistance to multi-lateralism on the one hand and the unviability of centralized control on the other (eg.^32^), such a polycentric system will require a balance between markets, government regulation and peer-to-peer commons-type institutional configurations.

Although transitions to what the Preamble to the Sustainable Development Goals (SDGs) referred to as a “transformed world” are being tackled from many different perspectives (see ref. ^33^), two mainstream schools of thought have emerged. Firstly, there is resilience thinking associated with the international Resilience Alliance and the Stockholm Resilience Centre. Here the focus is on regime shifts and transformations of social-ecological systems to cope with, adapt to, and transform in the face of change^30,34–36^. Secondly, there is the Dutch School of sustainability transition theory that emerged after the turn of the millennium^37–39^. Whereas resilience thinking’s roots are in ecosystem science^40^, the sustainability transitions approach emerged from evolutionary economics, science/technology and society studies, and innovations research. Both are premised on an interpretation of complexity theory and represent the foundation for the arguments presented here.

Box 1 Meta-governance Institution (e.g., an Ocean Agency) Although current agencies and multi-lateral institutions (such as UNEP, FAO, IMO) have validity and legitimacy^5,32^, progress towards a sustainable ocean is not in step with the pace of climate change and ocean degradations. Oceanic impacts are being experienced acutely among islands, coasts, fisheries and polar seas^144^. What is required is not “merely rearranging the organization of chairs on our planetary Titanic”^32^. Without fundamental change, the growth of the ocean economy is likely to exacerbate existing inequalities and accelerate the depletion of ocean resources and degradation of planetary environmental systems. Effective ocean polycentrism may therefore require a rule-setting global institution (or the restructuring of an existing global institution) to represent a common world view or value system, and to create good governance-inspired flexible frameworks for the implementation, monitoring and management of blue economy activities^5^. This global institution also would guide national policies and corporate activities, and manage disparate views and ideas of the multiple actors in ocean and coastal governance processes^145^. Governing the trade-offs between different policy objectives which will arise in multi-scalar, polycentric governance models will be easier if meta-governance principles such as transparency, accountability, and inclusivity are in place^3,146^. Without a shared set of norms, values and ‘rules of the game’, the bottom-up flourishing of commons initiatives will not have the systemic, transformative impact that is required. This meta-governance institution could be supported by a knowledge commons and mandated by states to create principled frameworks (eg. as in the UNESCO Man and Biosphere Program) to address ocean-related challenges at different scales, in response to changing needs, capacity and context (eg. the FAO Voluntary Guidelines for Securing Small Scale Fisheries).

## The dynamics of sustainable transitions and transformations

Since the beginning of human civilization, people have collaborated to secure and protect natural resources they have depended on for their survival^11^. Communities have routinely devised complex polycentric governance arrangements to transform open-access environments (such as fishing grounds) into regulated commons regimes^11,41^. Commons management of this nature is characterised by a commitment to equitable access, use and sustainability^42^.

During the process of industrialization, the commons has gradually been replaced by either private ownership or public goods owned or controlled by states^43,44^. Recent work to support sustainability transitions or transformative change in governance towards ecosystem stewardship has brought the commons back into focus, with a particular focus on natural systems, including forests, water resources, soils and the ocean^45^. Stewardship has been defined as a strategy to respond to and shape social-ecological systems under conditions of uncertainty and change to sustain both people and planet^44^. Stewardship approaches are designed to promote resilience when existing systems (socio-technical regimes, see Glossary in Table 1) are no longer able to accommodate or adapt to a new set of ecological, social or economic conditions^26,37–39,46^. Socio-technical regimes can reinforce entrenched governance arrangements that prevent effective responses to these landscape pressures. For example, despite the rapid growth in renewables worldwide, there is no significant decline in CO2 emissions, largely because of inter-dependent sets of interests reinforced by existing tax and subsidy regimes^47^. For a given socio-technical regime to give way to an alternative, a vast array of complex system components need to be dismantled and re-organized. Interests dependent on these socio-technical regimes often resist change. Resistance arises as a result of various factors including entrenched power relations, dominant economic or political subsystems, the limits of human imagination and societal norms^30^, or the failure to recognize that natural systems such as the ocean would not necessarily return to historically familiar conditions^48,49^.

**Fig. 1:**
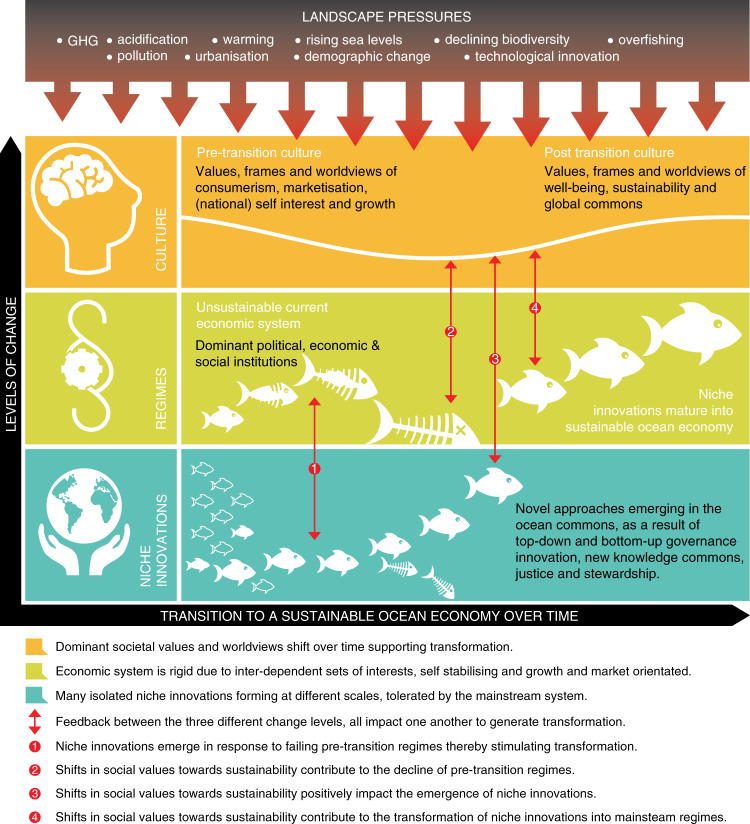
Dynamics of system-level change in the ocean economy. The elements of the ocean social-economic system undergoing systemic change as a result of interactions between culture, existing regimes, and niche innovations, all of which are influenced by landscape pressures. Redrawn from Narberhaus and Sheppard^116^, with permission from the author and in compliance with the CC BY-SA 3.0 license, 2015 https://creativecommons.org/licenses/by-nc-sa/3.0/.

How regimes respond to these pressures will depend on their internal capacity to manage change, and how they access new knowledge about alternatives. Without these conditions, regimes resist change and niche innovations can emerge. Such innovations are alternatives that respond to landscape pressures by challenging the logic and existence of a particular regime (e.g. local renewable energy systems in the face of non-responsive fossil fuel regimes) (see Glossary in Table 1), and which can, under certain conditions, catalyze pathways to transition. The dynamics of systemic change in the ocean economy are depicted in Fig. 1. The model represents the process of isolated, emergent niche innovations, some of which mature (or expand) and ultimately replace unsustainable political, economic and social institutions. This multi-level, dynamic process of transformation is influenced by increasing landscape pressures and informed by shifts in social culture towards a sustainable, commons-centric world view over time. (Conceptually this is similar to the notion of ‘seeds of the good anthropocene’^50^).

The starting point to define possible transition pathways to ocean stewardship is to (i) detail the primary drivers of change in the system, (ii) showcase demonstrations of how innovative leadership and niche-level experimentation in response to current drivers and incumbent regimes are beginning to shift the dynamics of transition, and (iii) highlight regime responses which have emerged. Although addressing the challenges facing the ocean system will require fundamental transformation, our explicit intention is to explore the evolutionary potential of the present to identify those experiments and change dynamics underway now that may aggregate into a much more significant transformative process in the future.

## Transition drivers and responses

Landscape pressures drive changes in the state of the environment, and impact parts of ecosystems or society. Rising levels of greenhouse gas emissions, and CO2 levels specifically, represent an overarching threat to a functional ocean^1,19^. Acidification is harming individual species and food webs, especially in the subarctic Pacific and western Arctic Ocean^2,20,51^. The ocean has absorbed over 90% of the excess heat from global warming, with consequences for the biogeography of species adapted to specific temperature ranges^21–23^. Declining oxygen content dramatically manifests in increases in extreme hypoxic events and species die-offs^24^, disrupting nearshore ecosystems and their dependent communities. Other primary landscape pressures on ocean systems include habitat destruction, overfishing, and run-off of pollution from land and coastal sources^1,25^ (Fig. 1).

In a reinforcing feedback loop, climate change and development pressures lead to changes in the ocean state (e.g., frequency of extreme storm events, food web structure), thereby increasing vulnerabilities and undermining key economic activities, such as fisheries and aquaculture, tourism and shipping^2^. Transitions to a more sustainable ocean may occur when current socio-political regimes confront landscape pressures they were not designed to handle. Responses by specific sectors or communities may generate innovations that, when combined and scaled, can contribute to enabling these transitions.

These niche innovations can be technical, cultural, social, economic, political or legal in nature—or a mixture of all—and provide examples of what is possible, thus catalysing further transition dynamics (Fig. 1). Several regime responses illustrate how shifts in existing ocean systems towards sustainability can be made possible. For example, the International Maritime Organization and the shipping industry have contributed to significant regime responses in the shipping sector, demonstrated by the regulation of green ship recycling^52–56^ and the decarbonisation of ocean transport^57^. In addition, fisheries certification schemes such as that developed and overseen by the Marine Stewardship Council have contributed to transparency, accountability and traceability in the ocean based food extraction regime^58^, but have also generated increased attention to what this certification scheme does not do, namely address social injustices arising from competitive but declining fisheries^59,60^. Finally, the agreement being negotiated under the provisions of UNCLOS, known as the internationally legally binding instrument for conservation and sustainable use of Biological Diversity in Areas Beyond National Jurisdiction, is intended to address challenges in marine biotechnology such as benefit sharing, area-based management tools, environmental impact assessments, capacity-building; and the transfer of marine technology^61^. We focus below on innovations that have emerged as responses to landscape pressures, in order to further demonstrate such transition dynamics.

Increased attention to ocean challenges and opportunities is generating a diversity of niche innovations in the ocean system. These innovations are distinctive in that they entail forms of stakeholder collaboration and co-development of solutions that are driven by an overriding concern to protect and regenerate the ocean commons. Niche innovations emerge beyond—but interact with—the regimes within which they occur, and they can eventually coalesce into alternative regimes (Table 2, Fig. 1, and Boxes 2–4). Existing regimes can engage with and absorb niche innovations as their way of responding and adapting to the perceived or real pressures. Sometimes these niches are too weak and are unable to generate alternative regimes, resulting in landscape breakdown and return to previous regimes^62^. In the right conditions, where the regime is accommodating, and the innovation is strong enough, a transition becomes possible (Fig. 1). To illustrate the breadth of niche innovations unfolding in the ocean system, we highlight six examples of how stakeholder communities are galvanizing around new approaches for ocean stewardship (Table 2). These are: (i) Integrated Ocean Management for development planning and disaster risk management through coastal zone planning (also see Box 2); (ii) rights-based fishery management in Chilean fisheries; (also see Box 3); (iii) pre-competitive collaboration and supply chain transparency in the seafood industry (also see Box 4); (iv) decarbonizing the maritime sector; (v) information sharing platforms for co-generation of knowledge and learning, and (vi) emerging legal approaches to the ocean commons.

**Table 2:** Examples of emerging niche innovations for ocean stewardship.

Approach	Pressures or impediments addressed	Description	Examples
i. Integrated Ocean Management for coastal zone development planning and disaster risk management	Uncoordinated ocean development; use conflicts across sectors; uneven and inequitable access to ocean resources, exposure to hazards, and available data/information	• A cross-sectoral approach which takes account of indirect, distant and cumulative impacts, recognizes trade-offs and uneven power relations between stakeholders • Offers lessons for durable outcomes and scaling transferable to anywhere in the world^119^ • In Belize (Box 2), diverse Ministries are collaborating and coordinating to develop a shared coastal zone vision across their many objectives (including disaster risk management), and to implement new policies and investments consistent with a cross-government Integrated Coastal Zone Management (ICZM) Plan. A new Ministry was formed from previous sector-based institutions to facilitate integration, and community groups (including private interests) helped both shape the vision for the ICZM Plan and also are instrumental in its implementation and monitoring. A rigorous science-policy engagement process conducted by the government is bringing rigor and transparency to a shared knowledge platform and implementation of the ICZM Plan. • As in Belize, governments are beginning to integrate disaster risk management with spatial development planning in coastal regions	*Belize* - Governance adaptation (Box 2). *Indonesia* - Ministry for Marine Affairs established *Fiji, Hawaii, EU* - Area-based and ‘ridge-to-reef’ management approaches for managing social -ecological systems^2,120,121^. *Barbados, Kenya, Seychelles* - ‘Blue Economy’ approaches *Vietnam, Phillipines, Indonesia, Fiji -* Disaster Risk Reduction in development planning and budgeting (United Nations’ Global Assessment on Disaster Risk Reduction)^122^
ii. Rights-based fishery management (RBFM)	Uneven and inequitable access to fisheries and livelihoods; lack of transparency in stock status, catch, and distribution of returns	• A collection of fishery management strategies: Territorial use rights for fishing (TURFs), individual transferable quotas (ITQs), and fishery cooperatives^58^ • Assigns exclusive rights to individual fishers, communities or cooperatives to harvest species based on a prescribed spatial area or catch limits • Can improve food security and livelihood support (especially in small-scale fisheries), and also economic returns (especially in larger, industrial fisheries); but explicit design for economic vs. social objectives is key^123,124^ • Monitoring and adaptive learning required for design^58,123^ • In Chile (Box 3), in response to declining coastal fisheries and livelihood support, an initial set of local fishing groups helped spur a governance transformation--a Territorial Use Rights in Fisheries (TURF) policy in 1991. Over the past 3 decades, capacity and political leverage of fisher associations have facilitated cross-scale and the cross- organizational interactions to help institutionalize the new governance regime. The evolving needs of the local TURFs, monitoring and scientific and legal analyses, have led to several governance amendments to improve the sustainability of the rights-based system over time.	*Chilean TURFs* (Box 3). *Pacific Islands’ Nauru Agreement* - cooperative fishing trading *Iceland ITQ fisheries*^125,126^ Added small boat ITQs in response to social concerns about sector consolidation in large commercial ITQ enterprises *Western Alaska Community Development Quota* – designed for rural communities^127^
iii. Pre-competitive collaboration and supply chain transparency	Declining fish stocks; unstable supply/value chains; inequitable distribution of financial and social impacts of fisheries; lack of transparency in impacts on shared ocean	• Improved transparency and oversight of supply chain mechanisms • Enables traceability for supply chain management in fisheries sector, informing consumer choice • Monitoring innovations including vessel monitoring systems (VMS) automatic identification systems (AIS) improving enforcement responses for illegal unreported and unregulated fishing • Supports justice in marine sustainability • Applicable to any ocean-dependent businesses • In the SeaBOS case (Box 4), 10 of the world’s largest seafood harvesting companies are managing seafood cooperatively, monitoring their practices and impacts, increasing transparency in supply chains and working together with governments to improve existing regulations concerning aquaculture and fisheries. The companies (encompassing hundreds of others in their value chain) are working outside their otherwise-competitive relationships and are collaborating with scientists to develop new approaches to managing their enterprises as stewards of the natural ocean assets upon which their businesses depend.	*SeaBOS* - Fishing industry initiative (Box 4) *Marine Stewardship Certification (MSC)* – labelling and certification *Abalobi app South Africa* – promotes traceable and storied seafood and supports small scale fishers *Conservation Alliance for Sustainable Seafood* – socially responsible seafood movement^128^ *Global Fishing Watch Platform* - real-time tracking of global commercial fishing activity via a public map^129^
iv. Decarbonising maritime sector	Increasing GHG emissions and environmental, social and political impacts	• The UN Climate Summit in September 2019 reiterated a global commitment to decarbonization^130^ • Commitments by many leading financial institutions to reduce investments in the oil sector • The International Chamber of Shipping (ICS) met in June 2019 to agree on actions in support of the IMO strategy to decarbonise shipping	*Norway & Washington* **–** electric ferries *Hong Kong harbor* **–** Solar-sail assisted ferries
v. Knowledge and information sharing platforms	Lack of transparency and access to data and information on the status/trends of ocean functions, resources & hazards, impacts of human activities and natural pressures, market pricing, and responses of ocean system to policy or investment interventions	• Open-source data and analytical platforms share information and learning in the ocean system^107^ • Opening access to more proprietary data sources will build trust and amplify information sharing • Used to design and improve content of information (peer-to-peer and at multiple scales by decision-making communities) • Catalyzing discussions with multiple actors across scales: how to standardize and improve data, analytics, and communication; tracking SDG progress and impacts of Nationally Determined Commitments (NDCs) under the UNFCCC • Facilitates clear signals for priority policy needs, and engagement between global and local scale actors • Can help accelerate these nascent efforts for adaptive governance, accountability and decision making at multiple scales • InVEST^131^ is driving integrated, multi-sectoral coastal development and disaster risk planning at national (Box 2) and regional scales around the world (e.g.^132–134^).	*InVEST platform* - Open source global data and software platform for quantifying ecosystem values^131^ *Ocean Data Interoperability Platform (ODIP)* – ocean data management and results sharing for EU, US, Australian providers *Ocean Biogeographic Information System (OBIS)* - world’s ocean biodiversity and biogeographic data and information sharing site; open access, to support UN and other policy efforts
vi. Legal Innovations (see also^30,135,136^)	Meta-governance and juridical principles for managing and protecting commons resources and spaces, limitations of national self-interest in state-centric system, securing environmental justice against polluters	• International measures to protect access and benefit sharing of marine resources in Areas Beyond National Jurisdiction (through for example establishment of MPAs, equitable sharing of benefits and access rights, guidelines for implementation of instruments into national policy and legislation) • Jurisprudence has recognized both procedural and substantive environmental rights • Earth system (or natural law) development allowing a previously unimaginable reality—rights for nature • An argument has emerged for a human right to an environment conducive to health and well-being and could contribute to environmental justice through the creation of a duty of care • Volitional reflexive governance (see Glossary in Table 1) to navigate the complexities of sovereignty and secure broad agreement on comprehensive rules for climate change and achieving the SDGs	*Meta-governance* **–** Biodiversity Beyond National Jurisdiction^61^ to be finalized in 2020, FAO Small Scale Fisheries Guidelines^91^, FAO Guidelines for implementation of international legal and policy instruments related to fisheries and conservation in Areas Beyond National Jurisdiction^90^, Organisation of African Unity Model Law^87^ *Jurisprudence* – Climate **(**eg.^137,138^), marine living resources (eg.^139^, enforceable rights for nature (eg.^140–142^) *International Agreements on Human Right to the Environment* (eg.^95,96,98–101,143^)(see also^144^) *Volitional Reflexive Governance* **-** UNFCCC Paris Agreement (see^85^), Voluntary National Review process under SDGs (see^70^).

These innovations are intended to be illustrative, and do not represent the full breadth of novel approaches surfacing around the world.

Despite these innovative responses, and emergent niches, key challenges in shifting dynamics to sustainable ocean governance remain, including a lack of coherence, coordination and clarity^27^, outdated regulatory assumptions^30,49^, conflict over allocation of space and rights of access to resources^63^, inadequate monitoring and enforcement (eg. ref. ^9^), lack of inclusivity (eg. ref. ^32^) and inequity in the distribution of ecosystem service benefits (eg. ref. ^5,64^). A purposeful shift towards governance for a sustainable ocean is required to address these challenges and allow the innovative approaches to emerge more fully.

Box 2 Coastal zone development planning in Belize The government of Belize’s Coastal Zone Act of 2000 recognizes the value of multi-sectoral, integrated spatial planning to guide policy and investment for more sustainable use of the coastal zone. The government approved a National Integrated Coastal Zone Management Plan (ICZMP) in 2016, led by a new Ministry inspired by the integrated development planning, connecting in one department Agriculture, Fisheries, Forestry, the Environment and Sustainable Development^147^. The plan was co-developed through an interactive stakeholder engagement process, beginning with identifying shared objectives for artisanal and commercial lobster and conch fisheries, reducing risk from sea-level rise and storms, and sustainable tourism benefits, the largest sector of the Belizean economy^148^. The final Plan is projected to achieve these goals through improved protection for mangroves, coral reefs, and seagrass beds^133,147,148^.

The final ICZMP highlights the importance of coordinating management of, and investment in, a diverse set of activities and actors, ranging from those affecting coastal pollution, dredging, fisheries, aquaculture, and tourism development, to education, social resilience to climate change, and preservation of cultural heritage. The plan led the Belizean government to enact a permanent ban for all oil exploitation within the second largest coral reef in the world. The ICZMP actions and new zoning-based management are being implemented with funding from the government, the Inter-American Development Bank, and other sources. The Belize Plan has been hailed by UNESCO as “one of the most forward-thinking ocean management plans in the world”^149^, and in 2017, UNESCO removed the Belize barrier reef from the World Heritage List in Danger because of the protections provided in the government ICZMP.

The key innovations in the Belize ICZMP process include a legal government mandate requiring a cross-sectoral spatial planning process. Such laws in and of themselves do not necessarily lead to transformation of ocean management. Belize’s Integrated Coastal Zone Management Authority and Institute (CZMAI) played a key role in designing the co-development process for the ICZMP, and continues to lead its ongoing implementation and adaptation. The science-policy process also included training of Belizeans on the scientific and policy aspects of Integrated Ocean Management, increasing the chances that the process will be internalized in government and civil society activities^148^.

Box 3 Chilean territorial use rights fisheries After an overfishing crisis led to critical closures of the Chilean abalone (“Loco”) fishery in the late 1980s, Chile enacted the first step in a governance transformation—a Territorial Use Rights in Fisheries (TURF) policy in 1991^150^. As of 2013, there are over 450 TURFs in full operation, making up >1100 km^2^ of subtidal habitat decreed to fisher organizations in Chile^153^. This network has been established by numerous associations of fishers under one policy instrument, Chile’s National Fisheries and Aquaculture Law^151,152^. As a result of the TURFs, Chile’s artisanal sector has increased in importance, with landings consistently surpassing the industrial catch since 2008. Artisanal fisheries are a significant source of employment for coastal communities, and their harvests represent a key source of nutritional food for many rural communities. Increases in biomass and size of individuals from species within properly managed TURFs also are demonstrating the potential of this rights-based management approach to sustain ecosystems and fishery benefits^151^.

The national enabling legislation, combined with the presence of scientific knowledge, and the capacity and political leverage of fisher associations who facilitated the cross- organizational interactions for change, each were key in institutionalizing the new governance regime. Participation in the program is voluntary, a key component of adaptive governance for a more resilient system. The TURF network has improved the knowledge of fishers and their access to learning and motivation for stewardship, especially as it relates to harvest management practices, biological aspects of the resource, and the interactions of the target species with other elements of the ecosystem.

While the 25-year old Chilean TURF model has proven its potential to improve the sustainability of fisher communities and fisheries, its governance must continue to evolve as information on social and ecological barriers to further scaling emerges^150^. TURFs convey rights to fishers and allow them a collective voice in the long-term management of the resource, a key component of their adaptability and responsiveness to changing social-ecological conditions. Currently, there is room for improvement with respect to enforcement, profitability, socioeconomic impacts on resource users, and the adaptability of the policy to local realities. Science is key to informing ways to maintain the policy, enable adaptation of TURFs and identify new conditions that must be improved for building resilience of TURFs or enable further transformations.

Box 4 Seafood business for ocean stewardship The Seafood Business for Ocean Stewardship (SeaBOS) initiative is an innovative collaboration among 10 of the largest global seafood companies that is transforming business operations for wild capture fisheries and aquaculture production. Collectively, this small group of 10 companies influences the strategic direction of more than 639 subsidiaries along the seafood value chain, with operations in at least 93 different countries, and participation in fisheries and aquaculture decision making institutions such as RFMOs. Under the SeaBOS platform, the world’s leading seafood businesses are managing seafood cooperatively, monitoring their practices and impacts, and charting a new path for their sector. They have pledged to address Illegal, Unreported and Unregulated (IUU) fishing; work towards full traceability and transparency throughout their supply chains; make efficient use of aquaculture feeds and fish feed resources from sustainably harvested stocks; apply existing certification standards; eradicate labour abuses and human rights violations from their supply chains; reduce the use of plastics in seafood operations; work towards reducing the use of antibiotics in aquaculture; and prevent harmful discharges and habitat destruction. The participating businesses also have pledged to work together with governments to improve existing regulations concerning aquaculture and fisheries^154^. The scope of the undertaking spans every continent and in all segments of seafood production. The collaborative nature of the SeaBOS project also helps companies share information to develop best practices which in turn, has helped to build trust and common purpose. An on-deck species-detecting camera and facial-image recognition software pilot is aimed at identifying illegal catch and undocumented fishermen on board vessels. SeaBOS has recognized the crucial role of scientists in framing the urgency of problems and potential solutions. The initiative is an on-going experiment that is being closely monitored to understand the significance of the changes over time. Such initiatives engaging with the private sector are best considered a complementary approach to existing processes, such as government regulations. This initiative is improving the prospects for transformative change by providing novel links between science and business, between wild-capture fisheries and aquaculture industries, and across geographical space^154^. SeaBOS is best described as a co-production initiative between science and business, in which companies can develop their agency^74^ to influence change, thereby contributing to amplifying new norms of ocean stewardship.

## Pathways for transition to a sustainable ocean economy

Conventional theories of change assume that political, social or market interventions can shift a system from one structure to another (such as the transition from feudalism to capitalism)^65^. These theories of change do not always apply for complex systems such as the ocean, where more incremental, learning-focused, and pragmatic approaches are more likely to lead to fundamental transformation^66^. Such transitions through incremental change depend on active learning based on real-time information. This is not necessarily a less radical option^65^.

**Fig. 2:**
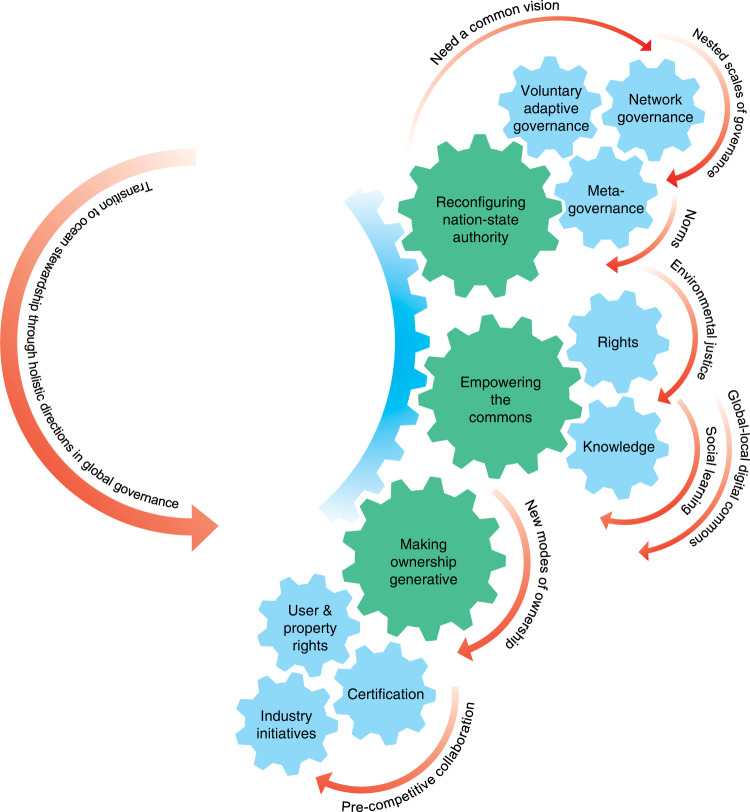
Elements of a governance transition to ocean stewardship. Elements informing a transition to a more adaptive and responsive global ocean governance system for ocean stewardship driven by three primary levers: reconfiguring nation state governance; empowering the commons through justice, equity and knowledge; and making ownership generative by integrating rights with responsibilities. Concepts based on Bollier^12^.

The governance mechanisms for managing both adaptive and transformative change require radical shifts if a more responsive global ocean governance system is to be established^10,30,67,68^. Reconfigured nation-state authority^69^ would occur by introducing principles such as reflexive, iterative governance (for example the voluntary national review process established for implementation and review of the UN Agenda 2020^70^) (Glossary in Table 1), by including polycentric modes of governance (nested scales of governance from local to national or global scales, demonstrated by the Belize and Chilean examples, Table 2, Boxes 2 and 3), and by creating meta-governance frameworks (such as the regime in Antarctica (Antarctic Treaty System^71^).  Empowered communities participate in decisions through legal rights as well as shared knowledge and information (Table 2). Finally, stewardship responsibilities should be integrated with user and property rights, and mainstreamed among corporations (evident in the SeaBOS example in Box 4*)*. The elements of a transition to global ocean governance are depicted in Fig. 2, and elaborated below.

A transformation to ocean stewardship is not a clear-cut, one-step change. Rather it is messy, fraught, contested, and occurs across different scales and domains^33,72^. Nevertheless, as we have shown, there are notable experiments and change dynamics already underway. The scale and scope of the contemporary transition required now is similar to that of the transition from hunter-gatherers to agrarian societies, and from agrarian societies to industrial societies^33,73^. In order to navigate the myriad complexities of this ocean transition, a balancing of economic, social and environmental objectives is required^5,10^. For example, the transformative approach of the United Nations’ 2030 Agenda and the Sustainable Development Goals (SDGs; specifically SDG 14—life below water) weaves together the numerous strands of sustainable development into an integrated set of ambitious goals that provides “coherence between policies and sectors, in all contexts—local, regional, national, transnational and global”^26^. It also provides a fundamental impulse for humanity—a common goal or a new “moon shot” for the global community^26^. The consistent and abiding reference point for the implementation of change is the protection of the commons. Stewardship of the commons has been repeatedly proven to work for millennia, while more recent Westphalian states and neoliberalism have arguably coincided with the age of unsustainability or the “early Anthropocene”^10^. To protect the commons, a reconfiguration of governance is required.

## Reconfiguring governance

Governance to meet the needs of bottom-up and top-down ocean stewardship processes will be most effective if it draws from three key frameworks: (1) polycentric, or network, governance; (2) voluntary, adaptive governance, and (3) meta-governance.

First, while there is a need to establish common legal principles, the diversity of the ocean commons also requires a system of flexible, adaptive governance to accommodate the interplay of actors with diverging interests^10,74^, involving diverse institutions, overarching rules, mutual adjustment, local action and trust building^30,75,76^. “Polycentric", or network governance can create a decentralized system of multiple self- governing and interacting groups at different scales across policy levels^45,69,77^, which often can handle complexity more effectively than centralized, top-down governance^78^. Transformation cannot rely entirely on bottom-up local initiatives—these will not necessarily “add up” to a coherent mode of ocean governance. However, successful self-governance of common pool resources by local communities studied by Ostrom^11^ demonstrates the viability of sustainable polycentric governance of natural resources beyond states and markets^3,78–81^ that function to recognise and design policy informed by site-specific preferences, competencies and the constraints of different actors^45^. This local tuning translates into reflexive and transformative governance, and can reconfigure the traditional socio-technical structures of nation-state authority so that they are more responsive to system changes.

Coordinated policy-making across borders and sectors is needed to implement shared goals for the ocean^5^. For example, the integrated planning in Belize (Box 2) allowed for interactions among public and private sector decision makers at multiple scales (local communities to government Ministers to the President). This resulted in distinct goals, policies, and actions for each of the nine different planning regions, that cumulatively created a consistent, shared national vision. Deeply engaged communities at the local level and an adaptive national government gave rise to a reconfigured, more integrated government ministry for improved coordination, and local leaders were empowered to nimbly adjust management of their natural resources to meet environmental, social and economic goals they had defined.

Critically, multi-scalar governance of natural resources can also focus attention on the way marginalized and vulnerable social groups already use, benefit and derive well-being from these resources^82^. For example, the declaration of a UNESCO World Heritage Site in Simangaliso in South Africa has disenfranchised a local community from a centuries old heritage of traditional fisheries usage rights, spiritual practices and customs as a result of the lack of authentic engagement with the local community^83^.

Second, voluntary commitments have become a well-recognized mechanism in international sustainability policy^84^. For example, the UNFCCC Paris Agreement includes voluntary commitments with sufficient flexibility to adapt to unfolding knowledge and system states and adjust to more ambitious goals, as well as obligatory procedural commitments such as transparency reporting^85^. Other examples of this style of “voluntary adaptive governance” include the Voluntary National Review process under the UN SDGs, and the Voluntary Commitments under the United Nations Our Ocean Conferences. In an evaluation of verifiable outcomes of voluntary commitments made at the Our Ocean Conferences from 2014 to 2017, Grorud-Colvert^86^ found that one third of the announcements focused on marine protected areas, and that almost half of these promised actions were completed at the time of publication. These voluntary commitments cumulatively added up to over 5 million km² of protected area, encompassing 1.4% of the ocean, almost doubling the quantity of implemented marine protected areas world-wide^86^.

Finally, to complement a flexible, polycentric approach to ocean governance, implementation in practice will need to be aligned with a measure of centralised “meta-governance” to collate information, design, and craft commons regulatory guidelines (Box 1). This central body would be responsible for developing best practices and establishing international guidelines for the implementation, monitoring and management of blue economy activities^5^. Several examples of supra-national frameworks exist in international environmental law. A regional example is the model law drafted by the Organisation for African Unity^87^, which provides a legislative guideline for domestic incorporation of the Convention on Biodiversity^88^, and the Nagoya Protocol^89^. Some established but innovative modes of supra-national governance for the ocean commons have been highly stable, such as the Antarctic Treaty System. The FAO Step-wise Guide for the implementation of international legal and policy instruments related to fisheries and conservation in areas beyond national jurisdiction^90^ is another example of this type of meta-governance instrument. The FAO guide offers a framework for incorporating international instruments pertaining to deep-sea fisheries and biodiversity conservation in the high seas into national policy and law. The guidance includes voluntary, flexible and practical guidelines for domestic legal integration, as well as overarching policy and normative guidelines. Other examples include numerous International Maritime Organisation Guidelines, the FAO Small Scale Fisheries Guidelines^91^, the Commonwealth Blue Charter, the European Commission’s Blue Growth Strategy and UNESCO’s Man and Biosphere framework. The latter, in particular, is a global governance framework whereby global ‘rules of the game’ for governance of a commons (biosphere reserves) are interpreted and implemented via local governance arrangements^92^.

## Empowering the commons through environmental justice and a shared knowledge commons

In order to ensure fairness and justice, compliance with environmental obligations is a matter of growing concern^3,77^. Stronger accountability, transparency and participation mechanisms will be required to clear conflicts and enable equitable sharing between different ocean users^93^. Compliance with environmental obligations is generally resolved at an inter-state level^94^, but if one accepts that the ocean is a global commons and is part of the global social-ecological system^1^, then in legal terminology, the obligations inherent in concepts such as the sustainable use of natural resources, inter-generational equity and the common concern of humankind fall into the category of obligations owed to the international community as a whole^94^. While commons have traditionally been held in trust by sovereign nations, or collaboratively managed through inter-state relationships, this has proven insufficient to protect the ocean and other planetary commons^10,81^.

The international recognition of an environmental human right through an international agreement^95–101^ discussed above would create an opportunity to generate a shift in the collective understanding of legal norms and environmental rights in a similar fashion to that which occurred in the human rights body of law as a result of the Universal Declaration of Human Rights six decades ago. This could initiate a paradigm shift in global culture towards a “human rights-based holistic environmental stewardship for the planet”. The FAO voluntary guidelines on natural resource tenure^102^ and on supporting small-scale fisheries^91^ are grounded in human rights principles, and are the first examples of such an approach being applied in fisheries policy. In addition to the embedded normative aspects of equity and justice, we argue that a human right to the environment would form a baseline “net” for governance of the ocean commons, through the inherent potential that such a right provides to address and redress the inequities suffered by individuals and communities exposed to environmental degradation and the unsustainable extraction of natural resources^103–105^.

A shared “information and learning commons” also will be a key feature supporting bottom-up governance processes. The internet has resulted in an entirely new global economy based on many-to-many interactions and vast data flows, fueling the rapid expansion of an information and knowledge commons. Information and communication technologies offer unprecedented potential for improving stewardship of ocean resources and ensuring resilient and productive ecosystems^106,107^. Yet existing databases are not readily accessible, and the compilation of data from ocean mapping, new marine and remote sensors and local knowledge is limited^106^. However, international efforts to address this are underway, such as the Ocean Infohub being developed under the auspices of the Intergovernmental Oceanographic Commission^106^. Such capability could be harnessed to reinforce a bottom-up ocean knowledge commons, enabling effective responses to climate- and development-related changes in the ocean^1,2^. Digitalisation hardly featured in the Paris Agreement or UN Agenda 2030, but it is increasingly clear that digital changes are becoming a key driving force in societal transformation, including for the ocean^108–111^. A shared information and knowledge commons would make “it possible to scale projects through new coordination mechanisms, which can allow small group dynamics to apply at the global scale. It is, thus, possible to combine ‘flatter’ structures and still operate efficiently on a planetary scale. This has never been the case before”^43^.

In this way. a digital ocean knowledge commons can evolve into the beating heart of a learning system which feeds back into and reinforces reflexive polycentric governance. Initially this would emerge from collaborations between existing research institutions who share a commitment to an open source knowledge commons. Calls for such a knowledge sharing function are emerging for ocean sectors (e.g. refs. ^106,107,112^), and an integrated information system would go a long way towards improved governance of the commons. Indeed, without this, there is no operating system to create a learning pattern into the future.

## Pre-competitive collaboration and property rights

The final building block of transformed governance to facilitate ocean stewardship is to make ownership generative (see Glossary in Table 1) by ensuring that market mechanisms, pre-competitive collaboration, property and usage rights are aligned with sustainability^113^. The concept of mobilizing corporations or integrating property rights with stewardship, embedded in this transition pathway, is already evident in the ocean economy.^63^. A substantial number of ocean industries have recently engaged in a process to advance pre-competitive collaboration for ocean stewardship, based on science, within the auspices of the United Nations Global Compact^106^. Although this initiative is still very new, it represents an important niche innovation with substantial potential for influencing the governance regime. Such pre-competitive collaboration between business actors and science is further illustrated by the SeaBOS example (Table 2 and Box 4). This initiative is based on a vision that a transition is possible. Participating companies share a definition of ocean stewardship as an adaptive and learning based, collaborative process of responsibility and ethics, aimed to shepherd and safeguard the resilience and sustainability of ocean ecosystems for human well-being. Fisheries management, in general, has seen a growing emphasis on the role, rights and responsibilities of small-scale fishers in stewardship of local resources^63^. Property rights are currently being integrated with stewardship around the world, as exemplified the Chilean TURF example described in Table 2 and Box 3.

## Conclusion: navigating the transition ahead

The ocean is a global commons. This statement now has very specific implications. The ocean, like the Earth’s atmosphere and soils, has made human civilization and life on Earth as we know it possible. However, like the atmosphere and soils, the ocean also faces fundamental threats that could lead to the collapse of critical biophysical functions and major societal disruption. A new system of global governance is required that responds to these pressures and recognizes the ocean as a global commons. Traditional nation-state and market-oriented governance mechanisms are not sufficient. Instead, we could build on a long tradition of polycentric governance arrangements for managing the commons that human civilizations developed long before the modern era of nation-states and markets. What is needed is a new mode of polycentric governance of the ocean-as-commons. This, however, cannot be imposed from above. It needs to build on the transition dynamics already underway at the niche and regime levels. It also must recognize the inherent complexity of the social-ecological ocean system, and facilitate nimble, rapid transformation through shared information and joint knowledge development.

A wide range of niche and regime responses have already arisen in response to the existence of landscape pressures, illustrating that a transition of some sort is already underway in the ocean. A purposeful transition would build on what is already emerging, and draw on a shared vision of ocean stewardship, incorporating a wide range of knowledge inputs, and accompanied by institutional capacity at all scales. This requires polycentric governance, transparency, legitimacy, and accountability, a well-resourced digital commons that connects shared data and learning with local innovations and applications; an environmental right to facilitate environmental justice for the ocean, and integration of stewardship responsibilities with rights to commons resources and spaces.

The current ocean governance system is insufficient for handling the challenges facing the ocean^10,114,115^, but there are options. We propose complementary mechanisms that would support and empower change. Further research is required to refine the alternatives, understand the complexities of niche- and regime-level innovations, and understand how promising examples can emerge and amplify the transition. History has demonstrated that social, economic and technological systems can and do transform, and that transitions can accelerate and generate impressive dynamics^115^. A reconfiguration of ocean governance should support a healthy and thriving relationship between humans and the ocean.
