# Author Correction: A transition to sustainable ocean governance

**DOI:** 10.1038/s41467-020-18409-5

**Published:** 2020-09-03

**Authors:** Tanya Brodie Rudolph, Mary Ruckelshaus, Mark Swilling, Edward H. Allison, Henrik Österblom, Stefan Gelcich, Philile Mbatha

**Affiliations:** 1 Centre for Complex Systems in Transition, University of Stellenbosch, Stellenbosch, South Africa; 2 The Natural Capital Project, Woods Institute for the Environment, Stanford University, Stanford, CA USA; 3 School of Public Leadership, University of Stellenbosch, Stellenbosch, South Africa; 4 Nippon Foundation Ocean Nexus Program, Earthlab, University of Washington, Seattle, WA USA; 5 WorldFish, Penang, Malaysia; 6 Stockholm Resilience Centre, Stockholm, Sweden; 7 Center of Applied Ecology and Sustainability, Department of Ecology, Pontificia Universidad Católica de Chile, Santiago, Chile; 8 Department of Environmental and Geographical Science, University of Cape Town, Cape Town, South Africa

**Keywords:** Ecology, Socioeconomic scenarios, Sustainability, Ocean sciences

Correction to: *Nature Communications* 10.1038/s41467-020-17410-2, published online 17 July 2020.

The original version of this Article omitted the following from the Acknowledgements: “This research is adapted from a Blue Paper commissioned by the High Level Panel for a Sustainable Ocean Economy entitled “The ocean transition: What to learn from system transitions” [https://www.oceanpanel.org/blue-papers/ocean-transition-what-learn-system-transitions].” This has now been corrected in both the PDF and HTML versions of the Article.

